# 6-Amino-4-(4-chloro­phen­yl)-2-oxo-1,2-dihydro­pyridine-3,5-dicarbonitrile ethanol solvate

**DOI:** 10.1107/S1600536808022551

**Published:** 2008-07-23

**Authors:** Runhong Jia, ShuJiang Tu

**Affiliations:** a Lianyungang Teacher’s College, Lianyungang 222006, People’s Republic of China; b College of Chemistry and Chemical Engineering, Xuzhou Normal University, Xuzhou 221116, People’s Republic of China

## Abstract

The title compound, C_13_H_7_ClN_4_O·C_2_H_6_O, was synthesized by the reaction of 4-chloro­benzaldehyde, malononitrile and 10% sodium hydroxide solution in an aqueous medium. In the crystal structure, the crystal packing is stabilized by inter­molecular N—H⋯N, O—H⋯O and N—H⋯O hydrogen bonds.

## Related literature

For related literature, see: Hasvold *et al.* (2003 ▶); Kappe (2004 ▶); Li *et al.* (2000 ▶); Mederski *et al.* (1999 ▶); Parlow *et al.* (2003 ▶); Varma (1999 ▶). 
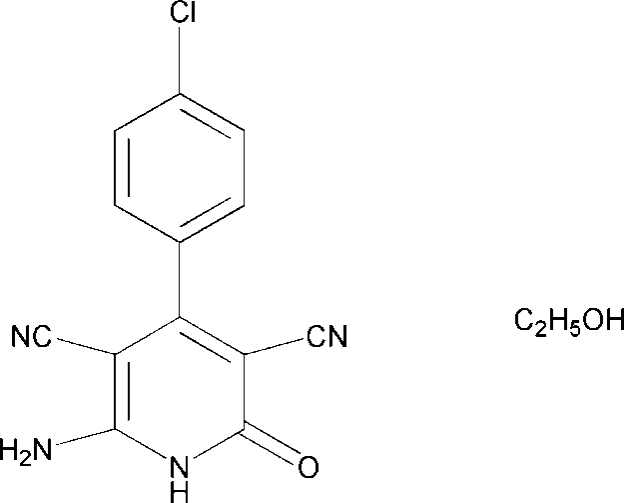


## Experimental

### 

#### Crystal data

C_13_H_7_ClN_4_O·C_2_H_6_O
*M* *_r_* = 316.74
Triclinic, 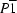

*a* = 6.7787 (10) Å
*b* = 10.4318 (14) Å
*c* = 11.2857 (17) Å
α = 88.634 (2)°
β = 84.643 (1)°
γ = 81.700 (1)°
*V* = 786.2 (2) Å^3^
*Z* = 2
Mo *K*α radiation
μ = 0.26 mm^−1^
*T* = 298 (2) K
0.14 × 0.09 × 0.03 mm

#### Data collection

Bruker SMART CCD area-detector diffractometer
Absorption correction: multi-scan (*SADABS*; Sheldrick, 1996 ▶) *T*_min_ = 0.965, *T*_max_ = 0.992
4165 measured reflections
2727 independent reflections
1162 reflections with *I* > 2σ(*I*)
*R*_int_ = 0.033

#### Refinement

*R*[*F* ^2^ > 2σ(*F* ^2^)] = 0.056
*wR*(*F* ^2^) = 0.140
*S* = 1.01
2727 reflections
199 parameters
H-atom parameters constrained
Δρ_max_ = 0.31 e Å^−3^
Δρ_min_ = −0.24 e Å^−3^

### 

Data collection: *SMART* (Bruker, 1998 ▶); cell refinement: *SMART*; data reduction: *SAINT* (Bruker, 1999 ▶); program(s) used to solve structure: *SHELXS97* (Sheldrick, 2008 ▶); program(s) used to refine structure: *SHELXL97* (Sheldrick, 2008 ▶); molecular graphics: *SHELXTL* (Sheldrick, 2008 ▶); software used to prepare material for publication: *SHELXTL*.

## Supplementary Material

Crystal structure: contains datablocks global, I. DOI: 10.1107/S1600536808022551/bq2082sup1.cif

Structure factors: contains datablocks I. DOI: 10.1107/S1600536808022551/bq2082Isup2.hkl

Additional supplementary materials:  crystallographic information; 3D view; checkCIF report

## Figures and Tables

**Table 1:** Hydrogen-bond geometry (Å, °)

*D*—H⋯*A*	*D*—H	H⋯*A*	*D*⋯*A*	*D*—H⋯*A*
O2—H2⋯O1^i^	0.82	2.02	2.755 (4)	149
N2—H2*B*⋯N3^ii^	0.86	2.25	3.084 (5)	164
N2—H2*A*⋯O2^iii^	0.86	1.98	2.832 (4)	168
N1—H1⋯O1^iv^	0.86	2.00	2.849 (4)	171

Symmetry codes: (i) 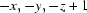; (ii) 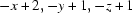; (iii) 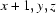; (iv) 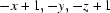.

**Fig. 1.**
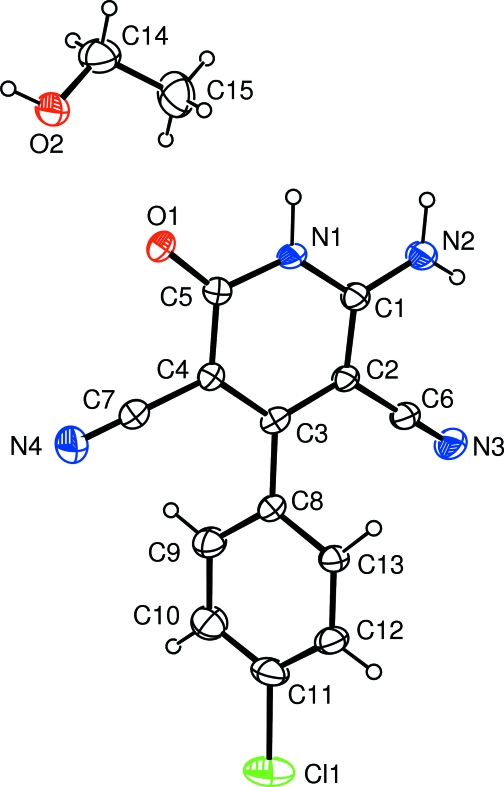
The molecular structure of title compound, showing 30% probability displacement ellipsoids.

**Fig. 2.**
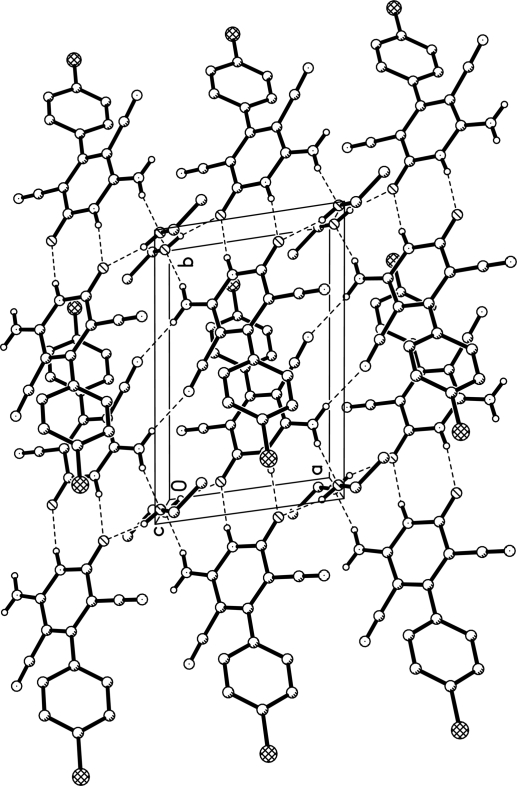
The packing diagram of title compound viewed along the a axis.

## supplementary crystallographic information

### Comment

In recent years, amino-substituted 2-pyridones have attracted attention due to their promising features as an important core structure for the development of biologically active molecules (Kappe, 2004). Pharmaceuticals with the 2- pyridone skeleton have emerged as antitumor (Varma, 1999), antifungal (Parlow *et al.*,2003), antibacterial (Hasvold *et al.* 2003), antiviral, antithrombotic (Li *et al.* 2000) agents. Meanwhile it is well known that the 2-pyridone ring system is a valuable building block in natural product synthesis. On the other hand, pyridine dicarbonitriles have been exhibited as potential novel prion disease therapeutics (Mederski *et al.* 1999). Therefore design and synthesis of these compounds has been challenging. For these reasons, the synthesis of compounds containing cyanopyridine derivatives is strongly desired. In this paper we report the crystal structure of the title compound, (I).

In the crystal structure, the dihedral angle between the C1/C2/C3/C4/C5/N1 plane and the C8—C13 benzene ring is 51.68 (13)° (Fig 1.). The molecules are connected *via* N—H···N and N—H···O intermolecular hydrogen bonds, forming a three-dimensional network (Table 1 and Fig. 2).

### Experimental

Compound (I) was prepared by the reaction of 4-chlorobenzaldehyde (1 mmol), malononitrile (2 mmol), 10% sodium hydroxide solution (1 ml) in water (2 ml). Single crystals of (I) suitable for X-ray diffraction were obtained by slow evaporation of a 95% aqueous ethanol solution (yield 48%; m.p. >573 K). IR (cm^-1^): 3450, 3317, 3205, 2216, 1669, 1590, 1484, 1378; ^1^H NMR (DMSO-d_6_): 7.53 (2*H*, d, J = 8.4 Hz, ArH), 7.64 (2*H*, d, J = 8.4 Hz, ArH), 7.72 (2*H*, brs, NH_2_), 11.94 (1*H*, s, NH).

### Refinement

All H atoms were positioned geometrically and treated as riding, with N—H = 0.86 Å, O–H = 0.82Å and C—H = 0.93–0.97 Å.

### Crystal data

**Table:**

C_13_H_7_ClN_4_O·C_2_H_6_O	*Z* = 2
*M**_r_* = 316.74	*F*_000_ = 328
Triclinic, *P*1	*D*_x_ = 1.338 Mg m^−^^3^
*a* = 6.7787 (10) Å	Mo *K*α radiation λ = 0.71073 Å
*b* = 10.4318 (14) Å	Cell parameters from 566 reflections
*c* = 11.2857 (17) Å	θ = 2.7–20.3º
α = 88.634 (2)º	µ = 0.26 mm^−^^1^
β = 84.6430 (10)º	*T* = 298 (2) K
γ = 81.7000 (10)º	Block, colorless
*V* = 786.2 (2) Å^3^	0.14 × 0.09 × 0.03 mm

### Data collection

**Table:**

Bruker SMART CCD area-detector diffractometer	2727 independent reflections
Radiation source: fine-focus sealed tube	1162 reflections with *I* > 2σ(*I*)
Monochromator: graphite	*R*_int_ = 0.033
*T* = 298(2) K	θ_max_ = 25.0º
φ and ω scans	θ_min_ = 2.0º
Absorption correction: multi-scan(SADABS; Sheldrick, 1996)	*h* = −8→7
*T*_min_ = 0.965, *T*_max_ = 0.992	*k* = −7→12
4165 measured reflections	*l* = −13→11

### Refinement

**Table:**

Refinement on *F*^2^	Secondary atom site location: difference Fourier map
Least-squares matrix: full	Hydrogen site location: inferred from neighbouring sites
*R*[*F*^2^ > 2σ(*F*^2^)] = 0.056	H-atom parameters constrained
*wR*(*F*^2^) = 0.140	*w* = 1/[σ^2^(*F*_o_^2^) + (0.0453*P*)^2^ + 0.0621*P*] where *P* = (*F*_o_^2^ + 2*F*_c_^2^)/3
*S* = 1.01	(Δ/σ)_max_ < 0.001
2727 reflections	Δρ_max_ = 0.31 e Å^−^^3^
199 parameters	Δρ_min_ = −0.24 e Å^−^^3^
Primary atom site location: structure-invariant direct methods	Extinction correction: none

### Special details

**Table:**

Geometry. All e.s.d.'s (except the e.s.d. in the dihedral angle between two l.s. planes) are estimated using the full covariance matrix. The cell e.s.d.'s are taken into account individually in the estimation of e.s.d.'s in distances, angles and torsion angles; correlations between e.s.d.'s in cell parameters are only used when they are defined by crystal symmetry. An approximate (isotropic) treatment of cell e.s.d.'s is used for estimating e.s.d.'s involving l.s. planes.
Refinement. Refinement of *F*^2^ against ALL reflections. The weighted *R*-factor *wR* and goodness of fit *S* are based on *F*^2^, conventional *R*-factors *R* are based on *F*, with *F* set to zero for negative *F*^2^. The threshold expression of *F*^2^ > σ(*F*^2^) is used only for calculating *R*-factors(gt) *etc*. and is not relevant to the choice of reflections for refinement. *R*-factors based on *F*^2^ are statistically about twice as large as those based on *F*, and *R*- factors based on ALL data will be even larger.

### Fractional atomic coordinates and isotropic or equivalent isotropic displacement parameters (Å^2^)

**Table:**

	*x*	*y*	*z*	*U*_iso_*/*U*_eq_	
Cl1	0.3974 (2)	0.82840 (13)	−0.02206 (12)	0.0905 (6)	
N1	0.5949 (5)	0.1640 (3)	0.4620 (3)	0.0420 (9)	
H1	0.6264	0.0964	0.5051	0.050*	
N2	0.8641 (5)	0.2447 (3)	0.5286 (3)	0.0520 (10)	
H2A	0.8877	0.1748	0.5700	0.062*	
H2B	0.9398	0.3040	0.5304	0.062*	
N3	0.8433 (5)	0.5684 (4)	0.4171 (3)	0.0565 (11)	
N4	0.0902 (6)	0.2779 (4)	0.1960 (3)	0.0705 (13)	
O1	0.3337 (4)	0.0727 (3)	0.4075 (2)	0.0544 (9)	
O2	−0.0003 (5)	0.0195 (3)	0.6598 (3)	0.0923 (13)	
H2	−0.0927	−0.0237	0.6639	0.138*	
C1	0.7115 (6)	0.2611 (4)	0.4621 (3)	0.0389 (11)	
C2	0.6615 (5)	0.3715 (4)	0.3915 (3)	0.0353 (10)	
C3	0.5067 (6)	0.3773 (4)	0.3158 (3)	0.0376 (10)	
C4	0.3897 (6)	0.2774 (4)	0.3224 (3)	0.0389 (11)	
C5	0.4310 (6)	0.1666 (4)	0.3979 (3)	0.0405 (11)	
C6	0.7651 (6)	0.4800 (4)	0.4034 (3)	0.0422 (11)	
C7	0.2249 (6)	0.2769 (4)	0.2514 (4)	0.0473 (12)	
C8	0.4755 (6)	0.4877 (4)	0.2317 (3)	0.0389 (10)	
C9	0.2890 (6)	0.5612 (4)	0.2250 (4)	0.0526 (12)	
H9	0.1789	0.5406	0.2733	0.063*	
C10	0.2668 (7)	0.6653 (5)	0.1464 (4)	0.0615 (14)	
H10	0.1416	0.7145	0.1427	0.074*	
C11	0.4283 (8)	0.6966 (4)	0.0736 (4)	0.0576 (13)	
C12	0.6122 (7)	0.6221 (4)	0.0768 (4)	0.0494 (12)	
H12	0.7202	0.6412	0.0259	0.059*	
C13	0.6369 (6)	0.5191 (4)	0.1552 (3)	0.0425 (11)	
H13	0.7622	0.4697	0.1574	0.051*	
C14	0.0839 (8)	0.0126 (5)	0.7684 (4)	0.0776 (16)	
H14A	0.1464	−0.0753	0.7828	0.093*	
H14B	−0.0200	0.0359	0.8324	0.093*	
C15	0.2369 (8)	0.1030 (5)	0.7664 (5)	0.0884 (18)	
H15A	0.2941	0.0980	0.8413	0.133*	
H15B	0.1743	0.1900	0.7528	0.133*	
H15C	0.3404	0.0788	0.7037	0.133*	

### Atomic displacement parameters (Å^2^)

**Table:**

	*U*^11^	*U*^22^	*U*^33^	*U*^12^	*U*^13^	*U*^23^
Cl1	0.1212 (13)	0.0645 (10)	0.0815 (10)	−0.0005 (8)	−0.0159 (8)	0.0352 (8)
N1	0.050 (2)	0.032 (2)	0.049 (2)	−0.0153 (18)	−0.0145 (17)	0.0133 (18)
N2	0.058 (2)	0.041 (2)	0.065 (2)	−0.0234 (19)	−0.025 (2)	0.015 (2)
N3	0.064 (3)	0.056 (3)	0.057 (2)	−0.028 (2)	−0.016 (2)	0.010 (2)
N4	0.063 (3)	0.068 (3)	0.088 (3)	−0.025 (2)	−0.031 (2)	0.018 (3)
O1	0.0521 (19)	0.047 (2)	0.074 (2)	−0.0301 (16)	−0.0224 (16)	0.0188 (17)
O2	0.097 (3)	0.081 (3)	0.119 (3)	−0.050 (2)	−0.067 (2)	0.045 (2)
C1	0.043 (3)	0.036 (3)	0.041 (2)	−0.011 (2)	−0.011 (2)	0.004 (2)
C2	0.041 (3)	0.031 (3)	0.037 (2)	−0.014 (2)	−0.0074 (19)	0.004 (2)
C3	0.041 (3)	0.034 (3)	0.038 (2)	−0.011 (2)	0.000 (2)	0.004 (2)
C4	0.038 (2)	0.040 (3)	0.042 (2)	−0.014 (2)	−0.008 (2)	0.008 (2)
C5	0.038 (3)	0.040 (3)	0.045 (3)	−0.007 (2)	−0.009 (2)	0.008 (2)
C6	0.047 (3)	0.041 (3)	0.041 (3)	−0.013 (2)	−0.006 (2)	0.007 (2)
C7	0.046 (3)	0.043 (3)	0.055 (3)	−0.015 (2)	−0.008 (2)	0.013 (2)
C8	0.040 (3)	0.034 (3)	0.045 (3)	−0.014 (2)	−0.005 (2)	0.007 (2)
C9	0.052 (3)	0.040 (3)	0.065 (3)	−0.005 (2)	−0.004 (2)	0.009 (3)
C10	0.052 (3)	0.050 (3)	0.077 (4)	0.006 (3)	−0.005 (3)	0.009 (3)
C11	0.072 (4)	0.045 (3)	0.057 (3)	−0.004 (3)	−0.018 (3)	0.016 (3)
C12	0.064 (3)	0.046 (3)	0.042 (3)	−0.023 (3)	−0.010 (2)	0.012 (2)
C13	0.043 (3)	0.042 (3)	0.045 (3)	−0.012 (2)	−0.010 (2)	0.008 (2)
C14	0.103 (4)	0.058 (4)	0.078 (4)	−0.023 (3)	−0.027 (3)	0.010 (3)
C15	0.085 (4)	0.074 (4)	0.115 (5)	−0.020 (3)	−0.036 (3)	−0.014 (4)

### Geometric parameters (Å, °)

**Table:**

Cl1—C11	1.728 (4)	C4—C7	1.435 (5)
N1—C1	1.372 (4)	C8—C9	1.388 (5)
N1—C5	1.377 (4)	C8—C13	1.401 (5)
N1—H1	0.8600	C9—C10	1.385 (5)
N2—C1	1.323 (4)	C9—H9	0.9300
N2—H2A	0.8600	C10—C11	1.380 (6)
N2—H2B	0.8600	C10—H10	0.9300
N3—C6	1.147 (5)	C11—C12	1.374 (6)
N4—C7	1.152 (5)	C12—C13	1.377 (5)
O1—C5	1.255 (4)	C12—H12	0.9300
O2—C14	1.396 (5)	C13—H13	0.9300
O2—H2	0.8200	C14—C15	1.497 (6)
C1—C2	1.404 (5)	C14—H14A	0.9700
C2—C3	1.407 (5)	C14—H14B	0.9700
C2—C6	1.432 (5)	C15—H15A	0.9600
C3—C4	1.395 (5)	C15—H15B	0.9600
C3—C8	1.479 (5)	C15—H15C	0.9600
C4—C5	1.429 (5)		
			
C1—N1—C5	124.9 (3)	C10—C9—C8	120.0 (4)
C1—N1—H1	117.5	C10—C9—H9	120.0
C5—N1—H1	117.5	C8—C9—H9	120.0
C1—N2—H2A	120.0	C11—C10—C9	120.7 (4)
C1—N2—H2B	120.0	C11—C10—H10	119.6
H2A—N2—H2B	120.0	C9—C10—H10	119.6
C14—O2—H2	109.5	C12—C11—C10	119.7 (4)
N2—C1—N1	118.0 (4)	C12—C11—Cl1	120.5 (4)
N2—C1—C2	124.1 (4)	C10—C11—Cl1	119.8 (4)
N1—C1—C2	117.9 (4)	C11—C12—C13	120.2 (4)
C1—C2—C3	120.7 (4)	C11—C12—H12	119.9
C1—C2—C6	118.1 (3)	C13—C12—H12	119.9
C3—C2—C6	121.1 (4)	C12—C13—C8	120.8 (4)
C4—C3—C2	118.4 (4)	C12—C13—H13	119.6
C4—C3—C8	122.5 (3)	C8—C13—H13	119.6
C2—C3—C8	119.1 (4)	O2—C14—C15	109.8 (4)
C3—C4—C5	121.8 (4)	O2—C14—H14A	109.7
C3—C4—C7	122.4 (4)	C15—C14—H14A	109.7
C5—C4—C7	115.7 (4)	O2—C14—H14B	109.7
O1—C5—N1	118.8 (4)	C15—C14—H14B	109.7
O1—C5—C4	125.3 (4)	H14A—C14—H14B	108.2
N1—C5—C4	115.9 (4)	C14—C15—H15A	109.5
N3—C6—C2	177.2 (5)	C14—C15—H15B	109.5
N4—C7—C4	178.7 (5)	H15A—C15—H15B	109.5
C9—C8—C13	118.5 (4)	C14—C15—H15C	109.5
C9—C8—C3	121.9 (4)	H15A—C15—H15C	109.5
C13—C8—C3	119.7 (4)	H15B—C15—H15C	109.5
			
C5—N1—C1—N2	179.8 (4)	C7—C4—C5—O1	−1.2 (6)
C5—N1—C1—C2	−0.3 (6)	C3—C4—C5—N1	−1.0 (6)
N2—C1—C2—C3	174.8 (4)	C7—C4—C5—N1	177.1 (4)
N1—C1—C2—C3	−5.0 (6)	C4—C3—C8—C9	52.2 (6)
N2—C1—C2—C6	−8.4 (6)	C2—C3—C8—C9	−128.5 (4)
N1—C1—C2—C6	171.8 (3)	C4—C3—C8—C13	−127.0 (4)
C1—C2—C3—C4	7.1 (6)	C2—C3—C8—C13	52.4 (5)
C6—C2—C3—C4	−169.6 (4)	C13—C8—C9—C10	−1.9 (6)
C1—C2—C3—C8	−172.3 (4)	C3—C8—C9—C10	178.9 (4)
C6—C2—C3—C8	11.0 (6)	C8—C9—C10—C11	0.4 (7)
C2—C3—C4—C5	−4.0 (6)	C9—C10—C11—C12	1.6 (7)
C8—C3—C4—C5	175.4 (4)	C9—C10—C11—Cl1	−179.0 (4)
C2—C3—C4—C7	178.1 (4)	C10—C11—C12—C13	−2.2 (7)
C8—C3—C4—C7	−2.5 (6)	Cl1—C11—C12—C13	178.5 (3)
C1—N1—C5—O1	−178.4 (4)	C11—C12—C13—C8	0.7 (6)
C1—N1—C5—C4	3.3 (6)	C9—C8—C13—C12	1.3 (6)
C3—C4—C5—O1	−179.2 (4)	C3—C8—C13—C12	−179.5 (4)

### Hydrogen-bond geometry (Å, °)

**Table:**

*D*—H···*A*	*D*—H	H···*A*	*D*···*A*	*D*—H···*A*
O2—H2···O1^i^	0.82	2.02	2.755 (4)	149
N2—H2B···N3^ii^	0.86	2.25	3.084 (5)	164
N2—H2A···O2^iii^	0.86	1.98	2.832 (4)	168
N1—H1···O1^iv^	0.86	2.00	2.849 (4)	171

Symmetry codes: (i) −*x*, −*y*, −*z*+1; (ii) −*x*+2, −*y*+1, −*z*+1; (iii) *x*+1, *y*, *z*; (iv) −*x*+1, −*y*, −*z*+1.
